# Bromodomain inhibitor I‐BET151 suppresses immune responses during fungal–immune interaction

**DOI:** 10.1002/eji.201848081

**Published:** 2019-06-21

**Authors:** Jorge Domínguez‐Andrés, Anaísa V Ferreira, Trees Jansen, Nicholas Smithers, Rab K. Prinjha, Rebecca C. Furze, Mihai G. Netea

**Affiliations:** ^1^ Department of Internal Medicine and Radboud Center for Infectious diseases (RCI) Radboud University Nijmegen Medical Centre 6500HB Nijmegen the Netherlands; ^2^ Instituto de Ciências Biomédicas Abel Salazar (ICBAS) Universidade do Porto 4050‐313 Porto Portugal; ^3^ Epigenetics DPU, Immuno‐Inflammation Therapy Area GlaxoSmithKline R&D, Medicines Research Centre Stevenage SG1 2NY UK; ^4^ Department for Genomics & Immunoregulation, Life and Medical Sciences Institute (LIMES) University of Bonn 53115 Bonn Germany; ^5^ Human Genomics Laboratory Craiova University of Medicine and Pharmacy Craiova Romania

**Keywords:** I‐BET151, *Aspergillus fumigatus*, *Candida albicans*, monocytes, neutrophils

## Abstract

Changes in the epigenetic landscape of immune cells are a crucial component of gene activation during the induction of inflammatory responses, therefore it has been hypothesized that epigenetic modulation could be employed to restore homeostasis in inflammatory scenarios. Fungal pathogens cause a large burden of morbidity and even mortality due to the hyperinflammatory processes that induce mucosal, allergic or systemic infections. Bromodomain and extraterminal domain (BET) proteins are considered as one as the most tantalizing pharmacological targets for the modulation of inflammatory responses at the epigenetic level. Nothing is known of the role of BET inhibitors on the inflammation induced by fungal pathogens. In the present study, we assessed the in vitro efficacy of the small molecular histone mimic BET inhibitor I‐BET151 to modulate innate immune responses during fungal–immune interaction with the clinically relevant fungal pathogens *Candida albicans* and *Aspergillus fumigatus*. Our results prove that BET inhibitors (I‐BETs) represent an important modulator of inflammation induced by fungal pathogens: both direct production of proinflammatory cytokines and the induction of trained immunity were inhibited by I‐BET151. These modulatory effects are likely to have important potential implications in clinically relevant situations.

## Graphical abstract

Treatment of immune cells with the clinically relevant small molecular histone mimic BET inhibitor I‐BET151 allows the modulation of immune responses during fungal–immune interaction through the reprogramming of the epigenetic landscape of the cells, decreasing the production of pro‐inflammatory cytokines and inhibiting the induction of trained immunity.

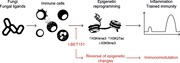

## Introduction

The incidence of fungal infections has risen dramatically over the past decade, with more than 2 million individuals suffering from life‐threatening systemic infections worldwide every year 1. These diseases are associated with a high mortality rate, exceeding 40% of the cases 2. Current therapies for invasive fungal infections, based on the use of antifungal drugs that target exclusively the fungus itself have low efficacy, and the emergence of resistance to antifungal agents is becoming a major concern 3. The design of effective therapies against fungal infections requires the development of new strategies that boost or optimize the actions of the host immune system, which could be combined with antifungal chemotherapy 1. One of the main mechanisms of pathogenicity during fungal infections is the development of excessive inflammation, due to the development of an exacerbated host immune innate response 4, 5. Thus, new therapies that modulate inflammatory responses during fungal diseases can contribute to improve the outcome of these infections.

Since enhanced inflammatory responses are related to altered epigenetic mechanisms, it has been hypothesized that epigenetic modulation could restore the normal balance in the immune system. In this regard, bromodomain and extraterminal domain (BET) proteins are considered to be promising pharmacological targets for the modulation of inflammatory responses at the epigenetic level. BET inhibitors (I‐BETs) can therefore be employed as modulators of the immune function and display important beneficial effects in clinically relevant scenarios. In addition, their use in a clinical context should also take into account their potential modulatory effects on infections often complicating inflammatory pathologies. Nothing is known on the role of BET inhibitors on the antifungal host response and the effects of fungal pathogens on inflammation. In the present study we report the in vitro efficacy of the clinically relevant small molecular histone mimic BET inhibitor I‐BET151 to modulate immune responses during fungal–immune interaction.

## Results and discussion

### I‐BET151 inhibits human monocyte, lymphocyte, and granulocyte functions after fungal stimulation

**Figure 1:**
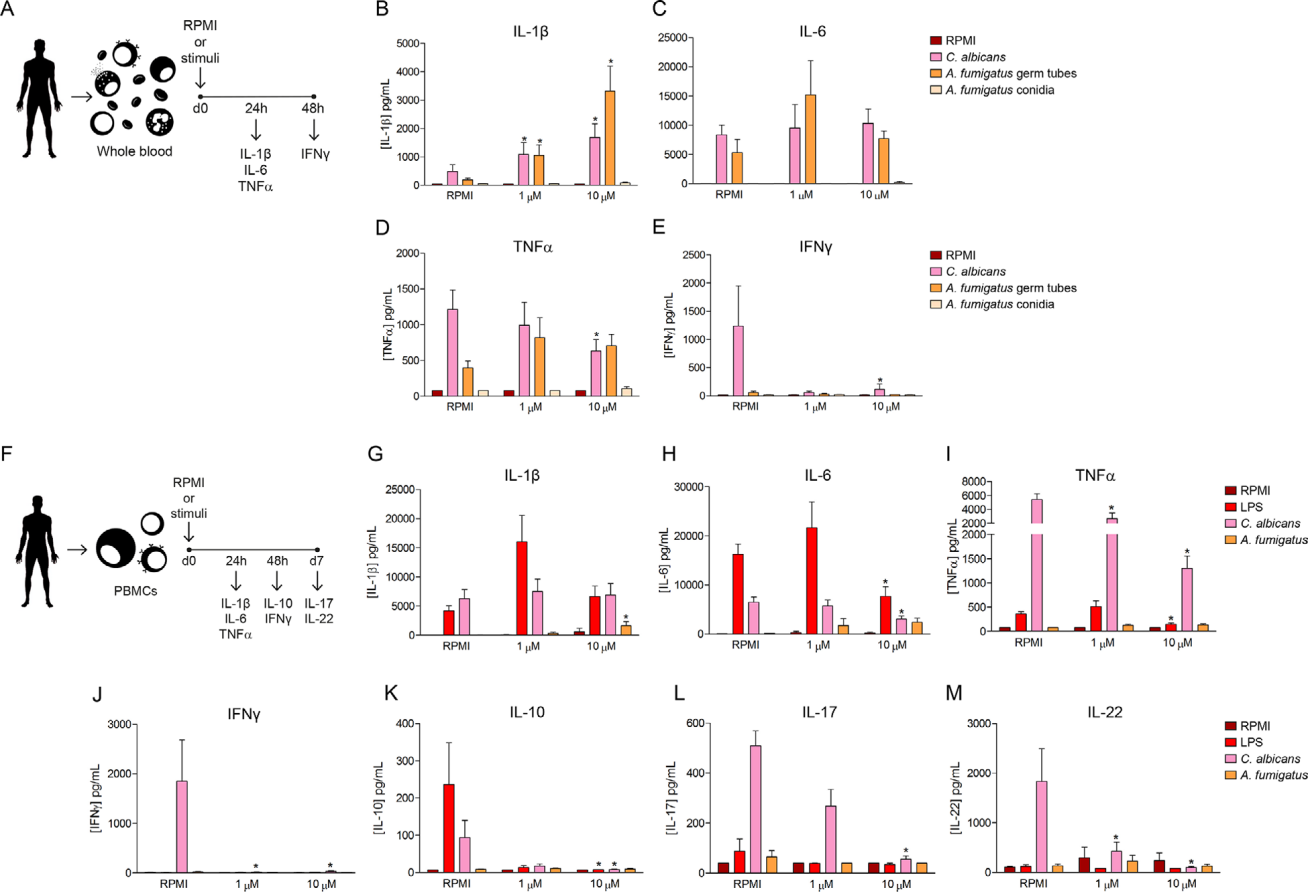
Cytokine production by WB cells and PBMCs. (A) Scheme of the procedure of WB stimulation. IL‐1β (B), IL‐6 (C), TNF‐α (D) and IFN‐γ (E) production by WB treated with medium, 10 or 1 µM I‐BET151 and further stimulated with RPMI, LPS, 10^6^ heat‐killed *C. albicans* conidia or 10^7^ heat‐killed *A. fumigatus* conidia. (F) Scheme of the procedure of PBMC stimulation. IL‐1β (G), IL‐6 (H), TNF‐α (I) and IL‐10 (J) production by human PBMCs treated with medium, 10 µM or 1 µM I‐BET151 and further stimulated with RPMI, LPS, 10^6^ heat‐killed *C. albicans* conidia or 10^7^ heat‐killed *A. fumigatus* conidia. IFN‐γ (K), IL‐17 (L) and IL‐22 (M) production by human PBMCs treated with medium, 10 µM or 1 µM I‐BET151 and further stimulated with RPMI, LPS, 10^6^ heat‐killed *C. albicans* conidia or 10^7^ heat‐killed *A. fumigatus* conidia. Mean ± SEM, *n* = 6 (from 6 different individual donors); pooled from 2 independent experiments with 3 individual donors each. *p < 0.05, Wilcoxon signed‐rank test.

We studied the influence of two different concentrations (1 and 10 µM, representing IC50 and IC100 for inhibition of LPS‐induced IL‐6 production of human blood assays; Supporting Information Fig. 1) of I‐BET151 in the cytokine production of whole blood (WB) cells after stimulation with heat‐killed forms of the clinically relevant fungal pathogens *Candida albicans* and *Aspergillus fumigatus* (Fig. 1A). The effects of I‐BET151 treatment on WB did not follow a consistent pattern, since the production of the pro‐inflammatory cytokine IL‐1β was significantly increased in a dose dependent way after *C. albicans* and *A. fumigatus* germ tube‐stimulation (Fig. 1B), while no changes were observed for IL‐6 production (Fig. 1C). A small inhibitory effect of I‐BET151 was observed on the production of TNF‐α (Fig. 1D). Of note, I‐BET151 was able to decrease the *Candida*‐induced production of the Th1‐derived cytokine IFN‐γ in the WB assay (Fig. 1E).

In the case of PBMC stimulation (Fig. 1F), the effects of I‐BET151 indicated a reduction in the production of cytokines induced by fungal stimulation. While I‐BET151 did not modify the production of IL‐1β induced by the fungal stimuli (Fig. 1G), the treatment with 10 µM of I‐BET151 led to a marked reduction of the production of the proinflammatory factors IL‐6 (Fig. 1H) and TNF‐α (Fig. 1I) after *C. albicans* and the control LPS stimulation. In addition, we also observed an inhibitory effect of I‐BET151 on the production of the anti‐inflammatory cytokine IL‐10, whose production was completely abolished by the treatment (Fig. 1J). In the same line, the *C. albicans*‐induced production of Th1 (IFN‐γ) and Th17 (IL‐17 and IL‐22) derived cytokines was strongly reduced by the treatment of cells with the inhibitor (Figs. 1K–M). This general decrease in cytokine production by PBMCs and monocytes after treatment with I‐BET151 is in line with the results shown by Nicodeme et al. in which they described the inhibition of the expression of pro‐inflammatory genes in macrophages treated with the compound 6.

**Figure 2:**
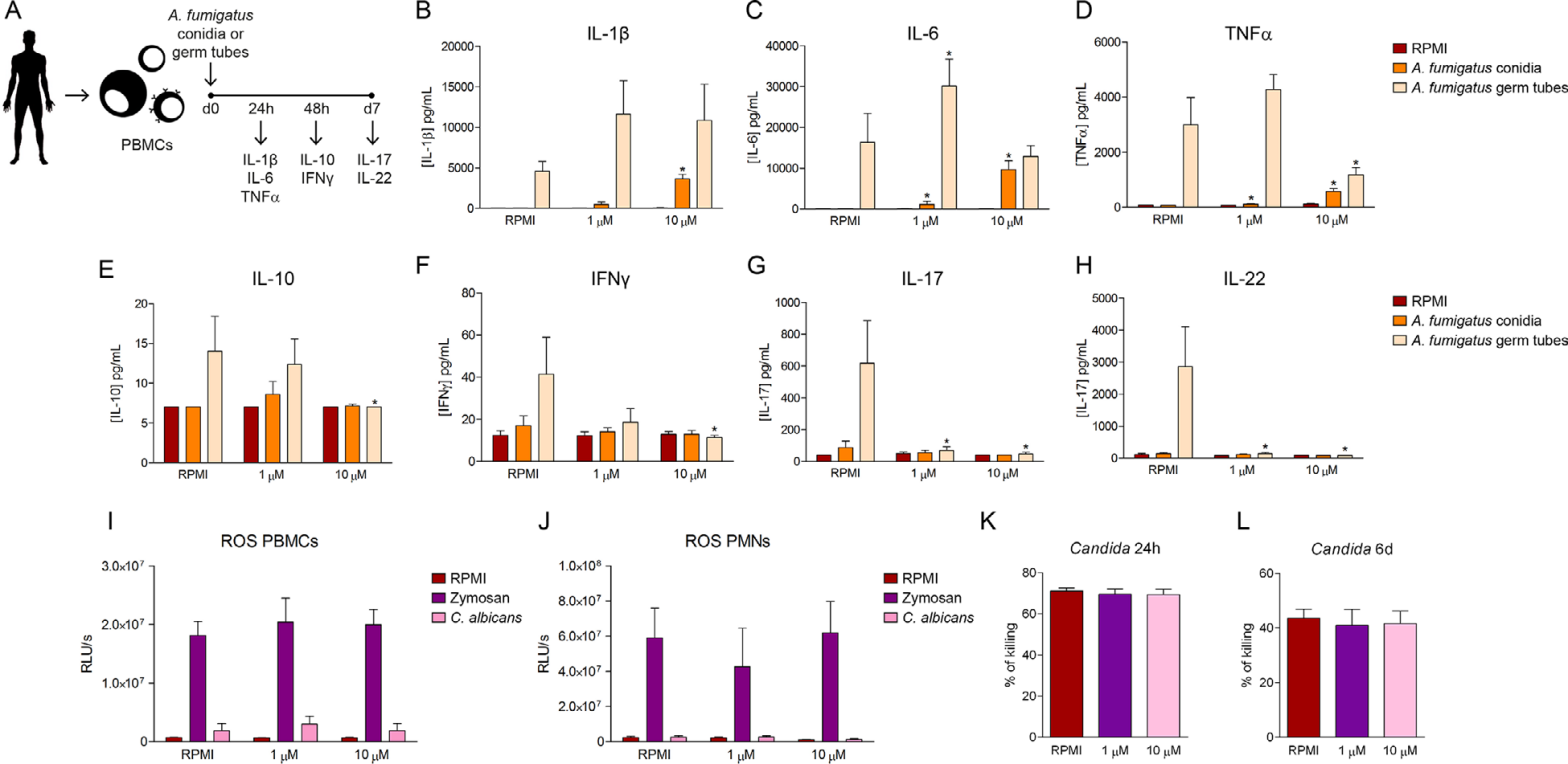
Fungal‐induced cytokine production by PBMCs. (A) Scheme of the procedure of PBMC stimulation. IL‐1β (B), IL‐6 (C), TNF‐α (D) and IL‐10 (E) production by PBMCs treated with medium, 1 µM or 10 µM I‐BET151 and further stimulated with RPMI, 10^7^ heat‐killed *A. fumigatus* conidia or 10^7^ heat‐killed *A. fumigatus* germ tubes. IFN‐γ (F), IL‐17 (G), and IL‐22 (H) production by PBMCs treated with medium, 10 or 1 µM I‐BET151 and further stimulated with RPMI, 10^7^ heat‐killed *A. fumigatus* conidia or 10^7^ heat‐killed *A. fumigatus* germ tubes. Mean ± SEM, *n* = 6 (from 6 different individual donors); pooled from two independent experiments with three individual donors each. *p < 0.05, Wilcoxon signed‐rank test. (I and J) ROS production of human PBMCs (I) or human neutrophils (J) treated with medium, 1 µM or 10 µM I‐BET151 after exposure to RPMI, 1 mg/mL Zymosan or 10^6^ heat‐killed *C. albicans* conidia (*n* = 4 (from 4 different individual donors); pooled from two independent experiments with two individual donors each). (K) % of *C. albicans* killing by human neutrophils (K) or monocytes (J) treated with medium, 10 µM or 1 µM I‐BET151 and further stimulated with RPMI or *C. albicans* conidia. (*n* = 6 (from 6 different individual donors); pooled from two independent experiments with three individual donors each).

Additionally, we studied the potential impact of the treatment with I‐BET151 in the different PBMC responses induced by of *A. fumigatus* conidia or *A. fumigatus* germ tubes (Fig. 2A), since they have been reported to induce different types of cytokine response 7. In this regard, the treatment with I‐BET151 led to a general reduction of the cytokine production induced by *A. fumigatus* conidia (Fig. 2B–D). The concentrations of IL‐10 induced by Aspergillus stimuli were low and thus no effects could be seen (Fig. 2E). Morbidity and mortality of aspergillosis can also be partly attributed to detrimental immune responses resulting from excessive adaptive immune activation 8. In this regard, we assessed that pre‐treatment of PBMCs with I‐BET151 led to a strong impairment of Th1 (IFN‐γ), and Th17‐ (IL‐17 and IL‐22) derived cytokine production induced after stimulation with germ tubes of *A. fumigatus* (Fig. 2F–H), highlighting the potential of this compound to counteract the harmful effects of exacerbated inflammation in fungal systemic infections. We also studied the possible influence of I‐BET151 treatment on ROS production by PBMCs (Fig. 2I) or neutrophils (Fig. 2J), as well as on the *Candida*‐killing abilities of neutrophils (Fig. 2H) and monocytes (Fig. 2I). No significant effects of I‐BET151 on these immune functions were identified.

### I‐BET151 impairs fungal‐induced trained immunity

**Figure 3:**
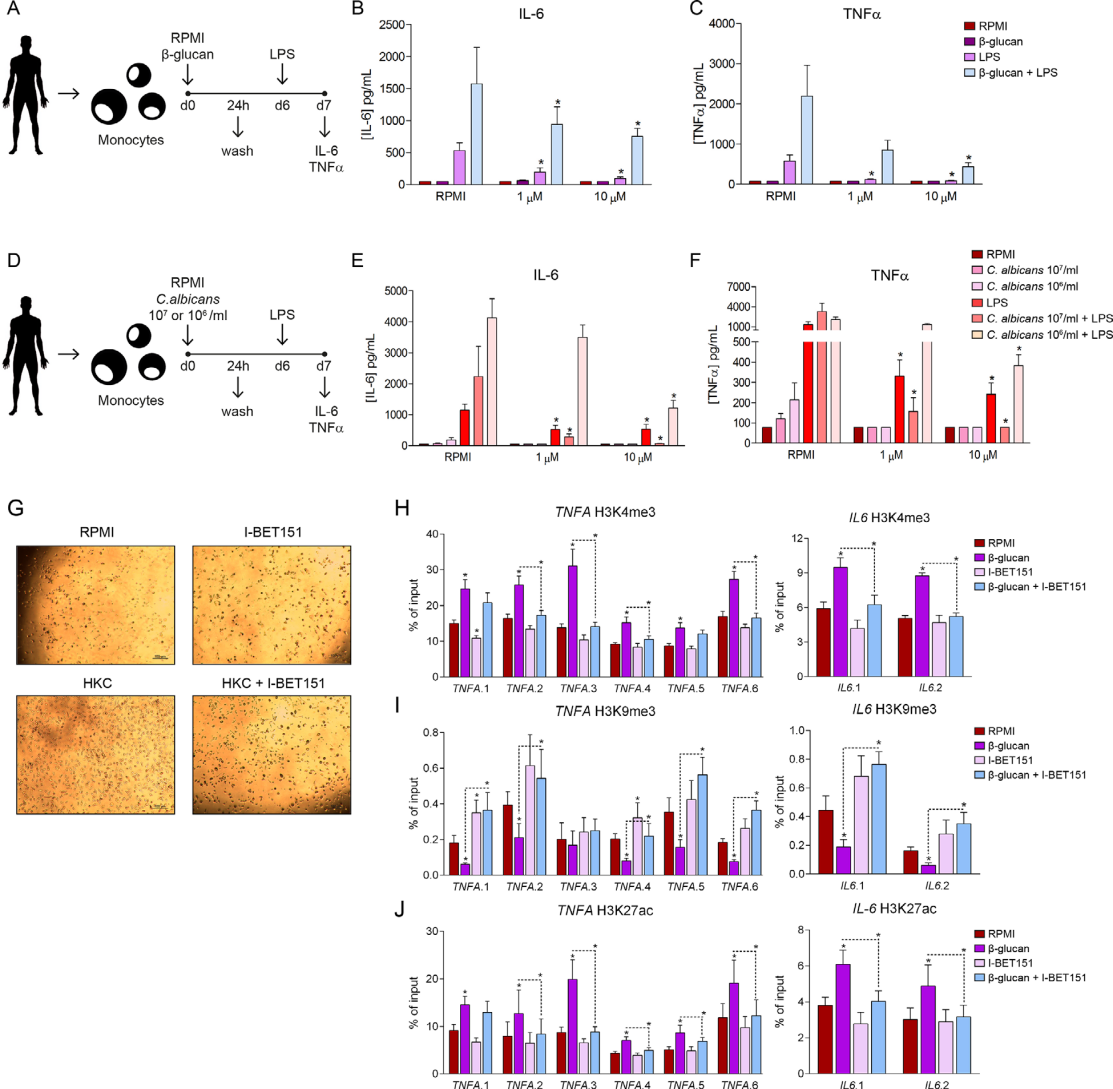
Effects of I‐BET151 in training of monocytes. (A) Scheme of the procedure followed in (B and C). IL‐6 (B) and TNF‐α (C) production by human monocytes treated with medium, 1 µM or 10 µM I‐BET151 and further stimulated with RPMI, 10 ng/mL LPS or 1 mg/mL β‐glucan for 24 h, followed by culture media. Where indicated, monocytes were re‐exposed to 10 ng/mL LPS after 6 days, and cytokines were measured 24 h later. (D) Scheme of the procedure followed in (E and F). IL‐6 (E) and TNF‐α production (F) by human monocytes treated or not with 10 or 1 µM I‐BET151 and stimulated with RPMI, 10^6^/mL or 10^7^/mL heat‐killed *C. albicans* conidia, or 10 ng/mL LPS followed by culture media. Where indicated, monocytes were re‐exposed to 10 ng/mL LPS after 6 days, and cytokines were measured 24 h later. (G) Morphology of cells after 24 h of training and 6 days of rest when the cells were trained with RPMI (negative control), 10 µM I‐BET151, 10^6^/mL heat‐killed *C. albicans* (HKC) or 10 µM I‐BET151 + 10^6^/mL heat‐killed *C. albicans*. Pictures were taken at day 6, magnification ×10. (H and I) Epigenetic effects of I‐BET151 treatment on β‐glucan‐trained monocytes. After 6 days of culture, H3K4me3 (H), H3K9me9 (I) and H3K27ac (J) marks were assessed at the level of promoters of *TNFA* and *IL6*. Mean ± SEM, *n* = 6 (from 6 different individual donors); pooled from two independent experiments with three individual donors each. *p < 0.05, Wilcoxon signed‐rank test.

Despite its central importance in host defense, inflammatory mechanisms are a double‐edged sword. A maintained induction of innate immune memory mechanisms might lead to development of chronic inflammatory conditions such as arthritis or atherosclerosis in the long term 9. Alternatively, inhibition of trained immunity could be used to counteract the excessive inflammation implicated in the pathogenesis of several diseases with an inflammatory component, such as diabetes 10, Alzheimer disease 11 or atherosclerosis 12. In this context, the development of new pharmacological strategies to counteract excessive and prolonged inflammation can be of crucial importance for the prevention and treatment of inflammation‐based diseases. To study the effects of I‐BET151 in innate immune reprogramming, we used a well‐established protocol of in vitro induction of trained immunity in monocytes 13, 14 (Fig. 3A). The treatment of these cells with I‐BET151 prior to the training with beta‐glucan led to a consistent decrease in the secondary LPS‐induced production of the cytokines IL‐6 and TNF‐α 13, 14 (Fig. 3B and C).

In order to study the specific effects of I‐BET151 in *Candida*‐induced trained immunity, we performed an experiment in which the monocytes were trained with different concentrations of heat‐killed *C. albicans* and the two concentrations of I‐BET151 tested in the previous experiments (Fig. 3D). As observed before, the treatment of cells with the inhibitor decreased the cytokine production induced by control LPS stimulation. Regarding the effects on *C. albicans*‐induced training, we observed a remarkable and dose‐dependent reduction in the induction of trained immunity in monocytes, as shown by the lower IL‐6 and TNF‐α levels produced by *C. albicans* trained cells after secondary stimulation by LPS (Fig. 3E and F). The differences observed in the induction of cytokine production between the two doses of *Candida* are most likely due to the tolerization effect of the high doses of stimuli on the cells 15. Upon training, the monocytes not only changed their response to secondary stimulation, but also their morphology. We analyzed cell morphology and cell size by using light microscopy. When cells were trained for 24 h and rested for 6 days, heat‐killed *C. albicans* (Fig. 3G) and β‐glucan (Supporting Information Fig. 3) induced the most remarkable changes in cell morphology: the cells were bigger than non‐trained cells. Treatment with I‐BET151 also influenced these morphological changes, as we observed only a slight increase in cell size compared to that for the untrained control cells and a clear decrease in size compared to HKC or β‐glucan‐trained cells (Fig. 3G and Supporting Information Fig. 3). Therefore, I‐BET151 showed powerful effects as an inhibitor of the induction of trained immunity in monocytes, both with β‐glucan and *C. albicans*, proving the large potential that this compound has as a pharmacological modulator of innate immune memory responses. Importantly, the results of the lactate dehydrogenase assay showed that the compounds were not inducing cell damage or cell death (Supporting Information Fig. 2A and B). Treatment of monocytes with the well‐known BET inhibitor JQ1 16 also decreased IL‐6 and TNF‐α production after induction of trained immunity with β‐glucan, but not with *Candida*. This confirms that the inhibition of BET proteins decreases the induction of inflammatory responses, and that the effects of I‐BET151 are stronger than those of other inhibitors of the same class (Supporting Information Fig. 4).

### I‐BET151 impairs fungal‐induced trained immunity at epigenetic level

Since epigenetic reprogramming plays a fundamental role in the induction of trained immunity 17, we checked the specific effects of the treatment with I‐BET151 on histone 3 lysine 4 trimethylation (H3K4me3) and histone H3K27 acetylation (H3K27ac), two epigenetic marks associated with increased gene transcription 18; and histone 3 lysine 9 trimethylation (H3K9me3), that has been well characterized as a mark of transcriptional repression 19. Both these marks have been reported to characterize the induction of trained immunity with β‐glucan in monocytes. In order to do that, we designed six different pairs of primers for the promoter region of *TNFA*, and two different pairs of primers for the promoter region of *IL6*. We assessed that the treatment with I‐BET151 inhibited the β‐glucan‐induced deposition of H3K4me3 and H3K27ac at the promoters of these proinflammatory cytokines (Fig. 3H and J). Conversely, it promoted the deposition of the repressor mark H3K9me3 (Fig. 3I), confirming the inhibitory role of I‐BET151 treatment in the induction of trained immunity at the epigenetic level. The analysis of the deposition of H3K4me3, H3K9me3 and H3K27ac marks in the promoter regions of the pro‐inflammatory cytokines involved in trained immunity reflects a scenario clearly repressive for inflammatory gene expression, supporting the role of I‐BET151 as an inhibitor of inflammation in the fungal–immune interactions.

### Concluding remarks

Here, we have assessed whether the bromodomain inhibitor I‐BET151 has the potential to modulate the responses of different innate immune cell subsets to fungal pathogens during acute stimulation, as well as in a model of trained immunity. I‐BET151 has previously been proven to be a very promising therapeutic approach to suppress inflammation in different contexts such as LPS‐induced endotoxic shock and bacteria‐induced sepsis 6, type I diabetes 20 or MLL‐fusion leukemia 21. The results presented here showed that treatment of human cells with the epigenetic modifier I‐BET151 decrease the inflammation induced by fungal stimulation both at the short and the long‐term. Of note, an appropriate production of cytokines such as IL‐17 or IFN‐γ is necessary to ensure an adequate immune response against fungal infections 22, 23. Therefore, we believe that I‐BET151 may be an exciting candidate to improve the treatment of patients suffering from exacerbated inflammatory response in the context of fungal infections. However, its effects should be monitored and the pharmacological doses employed strictly tested and further controlled in order to avoid an excessive dampening of the functions of the immune system. The data presented here open new lines of research and intervention for diseases with an inflammatory basis in which innate immune memory plays a central role, such as atherosclerosis, diabetes or autoimmune disorders 24.

## Materials and methods

### Peripheral blood mononuclear cell and monocyte isolation

Buffy coats from healthy donors were obtained after written informed consent (Sanquin Blood Bank, Nijmegen, the Netherlands). Samples were anonymized to safeguard donor privacy. PBMC isolation was performed by differential density centrifugation over Ficoll‐Paque (GE Healthcare). Percoll isolation of monocytes was performed as previously described 25. Briefly, 150–200 × 10^6^ PBMCs were layered on top of a hyper‐osmotic Percoll solution and centrifuged for 15 min at 580 × *g*. The interphase layer was isolated and cells were washed with cold PBS. Cells were re‐suspended in RPMI medium Dutch modified (Invitrogen) supplemented with 50 µg/mL gentamicin, 2 mM Glutamax, and 1 mM pyruvate, and counted. An extra purification step was added by adhering Percoll‐isolated monocytes to polystyrene flat bottom plates (Corning) for 1 h at 37˚C; a washing step with warm PBS was then performed to yield maximal purity.

### Stimulation and trained immunity experiments

5 × 10^5^ PBMCs/mL or 10^5^ monocytes were added to flat‐bottom 96‐well plates (Greiner). Cells were stimulated with RPMI, heat‐killed 10^7^/mL *A. fumigatus* germ tubes, 10^7^/mL *A. fumigatus* conidia, 10^6^/mL or 10^7^/mL heat killed *C. albicans* yeast, 10 ng/mL *E. coli* LPS (serotype 055:B5, Sigma‐Aldrich), 1 µg/mL β‐glucan (β‐1,3‐(D)‐glucan, kindly provided by Professor David Williams), 10 µM I‐BET151, 0.05 µM JQ1 (based on the results of the cytotoxicity assay shown in Supporting Information Fig. 4A), or 1 µg/mL β‐glucan + 10 µM I‐BET151, and restimulated after 6 days with 10 ng/mL *E. coli* LPS. Supernatants from monocytes were collected 24 h after stimulation. Cells were washed once with 200 µL warm PBS and incubated for 5 days in culture medium with 10% serum and medium was changed once. After 24 h, supernatants were collected and stored at −20°C.

### Cytokine measurements

Cytokine production from human cells was determined in supernatants using commercial ELISA kits for IL‐1β, IL‐6, TNF‐α, IL‐10, IFN‐γ, IL‐17, and IL‐22 (R&D Systems, Minneapolis, MN, USA) following the instructions of the manufacturer.

### Chromatin immunoprecipitation

Purified cells were fixed with 1% formaldehyde (Sigma) at a concentration of approximately 10^6^ cells/mL. Fixed cell preparations were sonicated using a Diagenode Bioruptor UCD‐300 for 3 × 10 min (30 s on; 30 s off). 67 mL of chromatin (one million cells) was incubated with 229 mL dilution buffer, 3 mL protease inhibitor cocktail and 0.5−1 mg of H3K4me3, H3K9me3, or H3K27Ac antibodies (Diagenode) and incubated overnight at 4°C with rotation. Protein A/G magnetic beads were washed in dilution buffer with 0.15% SDS and 0.1% BSA, added to the chromatin/antibody mix and rotated for 60 min at 4°C. Beads were washed with 400 mL buffer for 5 min at 4C with five rounds of washes. After washing chromatin was eluted using elution buffer for 20 min. Supernatant was collected, 8 mL 5M NaCl, 3 mL proteinase K were added and samples were incubated for 4 h at 65°C. Finally, samples were purified using QIAGEN; Qiaquick MinElute PCR purification Kit and eluted in 20 mL EB. Primer pairs employed for quantitative PCR can be found in Table 1.

**Table 1:** Primer pairs used for epigenetic study

*IL6.1*	FW	TCGTGCATGACTTCAGCTTT
*IL6.1*	RV	GCGCTAAGAAGCAGAACCAC
*IL6.2*	FW	AGGGAGAGCCAGAACACAGA
*IL6.2*	RV	GAGTTTCCTCTGACTCCATCG
*TNFA.1*	FW	AGAGGACCAGCTAAGAGGGA
*TNFA.1*	RV	AGCTTGTCAGGGGATGTGG
*TNFA.2*	FW	GTGCTTGTTCCTCAGCCTCT
*TNFA.2*	RV	ATCACTCCAAAGTGCAGCAG
*TNFA.3*	FW	TGTCTGGCACACAGAAGACA
*TNFA.3*	RV	CCCTGAGGTGTCTGGTTTTC
*TNFA.4*	FW	AGCCAGCTGTTCCTCCTTTA
*TNFA.4*	RV	TTAGAGAGAGGTCCCTGGGG
*TNFA.5*	FW	TGATGGTAGGCAGAACTTGG
*TNFA.5*	RV	ACTAAGGCCTGTGCTGTTCC
*TNFA.6*	FW	CAGGCAGGTTCTCTTCCTCT
*TNFA.6*	RV	GCTTTCAGTGCTCATGGTGT

### ROS production

Oxygen radical production levels were evaluated using luminol‐enhanced chemiluminescence and determined in a luminometer. Cells (1 × 10^5^ per well) were incubated in medium containing either RPMI, heat‐killed Candida (10^7^ yeast cells) or opsonized zymosan (1 mg/mL). Luminol was added to each well in order to start the chemiluminescence reaction. Each measurement was carried out in at least duplicate repetitions. Chemiluminescence was determined every 145 s at 37˚C for 1 h. Luminescence was expressed as relative light units (RLU) per second.

### Statistical analysis

Data are presented as mean ± SEM, as indicated in the legend of each figure, unless otherwise stated. The significance of the differences between groups was evaluated using Wilcoxon signed‐rank test. Data are judged to be statistically significant when p < 0.05 by two‐tailed Student's *t*‐test. In figs, asterisks denote statistical significance (*p < 0.05; **p < 0.01; ***p < 0.001).

### Fungal killing assay

*C. albicans* conidia were exposed to neutrophils or monocytes for 2 h in a 2:1 yeast‐to‐immune cell ratio. Neutrophils and monocytes were then lysed with Triton X‐100 solution 0.5% and the number of surviving yeast cells were assessed on Sabouraud agar plates. Killing activity was expressed as percentage of yeast surviving in the presence of monocytes compared to that surviving in the absence of monocytes.

## Conflict of interest

R.F., N.S., and R.K.P. are employees and shareholders at GSK, which is progressing BET protein inhibitors through clinical development. J.D.‐A., T.J., A.F., and M.G.N. do not have anything to disclose regarding funding or conflicts of interest with respect to this manuscript.

**Abbreviations**
BET: bromodomain and extraterminal domain
H3K27ac: histone H3K27 acetylation
H3K4me3: histone 3 lysine 4 trimethylation
I‐BET: BET inhibitor
RLU: relative light unit
WB: whole blood

## Supporting information

Figure S1. Target engagement plots for GSK1210151A (I‐BET151).
Figure S2. Cytotoxicity assay for I‐BET151 treatment.
Figure S3. Morphological changes induced by trained immunity.
Figure S4. Effects of JQ1 in the induction of trained immunity.
Table 1. Primer pairs used for epigenetic study.

## References

[1] Brown, G. D. , Denning, D. W. , Gow, N. A. R. , Levitz, S. M. , Netea, M. G. and White, T. C. , Hidden killers: human fungal infections. Sci. Transl. Med. 2012; 4. 10.1126/scitranslmed.3004404 23253612

[2] Kullberg, B. J. and Arendrup, M. C. , Invasive candidiasis. N. Engl. J. Med. 2015; 373: 1445–1456. 26444731 10.1056/NEJMra1315399

[3] Kanafani, Z. A. and Perfect, J. R. , Antimicrobial resistance: resistance to antifungal agents: mechanisms and clinical impact. Clin. Infect. Dis. 2008; 46: 120–128. 18171227 10.1086/524071

[4] Majer, O. , Bourgeois, C. , Zwolanek, F. , Lassnig, C. , Kerjaschki, D. , Mack, M. , Müller, M. et al., Type I interferons promote fatal immunopathology by regulating inflammatory monocytes and neutrophils during *Candida* infections. PLoS Pathog. 2012; 8: e1002811. 22911155 10.1371/journal.ppat.1002811 PMC3406095

[5] Lionakis, M. S. , Swamydas, M. , Fischer, B. G. , Plantinga, T. S. , Johnson, M. D. , Jaeger, M. , Green, N. M. et al., CX3CR1‐dependent renal macrophage survival promotes *Candida* control and host survival. J. Clin. Invest. 2013; 123: 5035–5051. 24177428 10.1172/JCI71307 PMC3859390

[6] Nicodeme, E. , Jeffrey, K. L. , Schaefer, U. , Beinke, S. , Dewell, S. , Chung, C. , Chandwani, R. et al., Suppression of inflammation by a synthetic histone mimic. Nature 2010; 468: 1119–1123. 21068722 10.1038/nature09589 PMC5415086

[7] Chai, L. Y. A. , Vonk, A. G. , Kullberg, B. J. , Verweij, P. E. , Verschueren, I. , van der Meer, J. W. M. , Joosten, L. A. B. et al., *Aspergillus fumigatus* cell wall components differentially modulate host TLR2 and TLR4 responses. Microbes Infect. 2011; 13: 151–159. 20971208 10.1016/j.micinf.2010.10.005

[8] Dewi, I. M. W. , van de Veerdonk, F. L. and Gresnigt, M. S. , The multifaceted role of T‐helper responses in host defense against *Aspergillus fumigatus* . J. Fungi (Basel) 2017; 3. 10.3390/jof3040055 PMC5753157 29371571

[9] Christ, A. , Bekkering, S. , Latz, E. and Riksen, N. P. , Long‐term activation of the innate immune system in atherosclerosis. Semin. Immunol. 2016; 28: 384–393. 27113267 10.1016/j.smim.2016.04.004

[10] Boutens, L. , Hooiveld, G. J. , Dhingra, S. , Cramer, R. A. , Netea, M. G. and Stienstra, R. , Unique metabolic activation of adipose tissue macrophages in obesity promotes inflammatory responses. Diabetologia 2018; 61: 942–953. 29333574 10.1007/s00125-017-4526-6 PMC6448980

[11] Wendeln, A.‐C. , Degenhardt, K. , Kaurani, L. , Gertig, M. , Ulas, T. , Jain, G. , Wagner, J. et al., Innate immune memory in the brain shapes neurological disease hallmarks. Nature 2018: 1. 10.1038/s41586-018-0023-4 PMC6038912 29643512

[12] Christ, A. , Günther, P. , Lauterbach, M. A. R. , Duewell, P. , Biswas, D. , Pelka, K. , Scholz, C. J. et al., Western diet triggers NLRP3‐dependent innate immune reprogramming. Cell 2018; 172: 162–175.e14. 29328911 10.1016/j.cell.2017.12.013 PMC6324559

[13] Bekkering, S. , Blok, B. A. , Joosten, L. A. B. , Riksen, N. P. , van Crevel, R. and Netea, M. G. , In vitro experimental model of trained innate immunity in human primary monocytes. Clin. Vaccine Immunol. 2016; 23: 926–933. 27733422 10.1128/CVI.00349-16 PMC5139603

[14] Saeed, S. , Quintin, J. , Kerstens, H. H. D. , Rao, N. A. , Aghajanirefah, A. , Matarese, F. , Cheng, S.‐C. et al., Epigenetic programming of monocyte‐to‐macrophage differentiation and trained innate immunity. Science 2014; 345: 1251086. 25258085 10.1126/science.1251086 PMC4242194

[15] Ifrim, D. C. , Quintin, J. , Joosten, L. A. B. , Jacobs, C. , Jansen, T. , Jacobs, L. , Gow, N. A. R. et al., Trained immunity or tolerance: opposing functional programs induced in human monocytes after engagement of various pattern recognition receptors. Clin. Vaccine Immunol. 2014; 21: 534–545. 24521784 10.1128/CVI.00688-13 PMC3993125

[16] Mietton, F. , Ferri, E. , Champleboux, M. , Zala, N. , Maubon, D. , Zhou, Y. , Harbut, M. et al., Selective BET bromodomain inhibition as an antifungal therapeutic strategy. Nat. Commun. 2017; 8: 15482. 28516956 10.1038/ncomms15482 PMC5454392

[17] van der Heijden, C. D. C. C. , Noz, M. P. , Joosten, L. A. B. , Netea, M. G. , Riksen, N. P. and Keating, S. T. , Epigenetics and trained immunity. Antioxid. Redox Signal. 2017:ars.2017.7310. 10.1089/ars.2017.7310 PMC6121175 28978221

[18] Kusch, T. , Histone H3 lysine 4 methylation revisited. Transcription 2012; 3: 310–314. 23117820 10.4161/trns.21911 PMC3630187

[19] Foret, M. R. , Sandstrom, R. S. , Rhodes, C. T. , Wang, Y. , Berger, M. S. and Lin, C.‐H. A. , Molecular targets of chromatin repressive mark H3K9me3 in primate progenitor cells within adult neurogenic niches. Front. Genet. 2014; 5: 252. 25126093 10.3389/fgene.2014.00252 PMC4115620

[20] Fu, W. , Farache, J. , Clardy, S. M. , Hattori, K. , Mander, P. , Lee, K. , Rioja, I. et al., Epigenetic modulation of type‐1 diabetes via a dual effect on pancreatic macrophages and β cells. Elife 2014; 3: e04631. 25407682 10.7554/eLife.04631 PMC4270084

[21] Dawson, M. A. , Prinjha, R. K. , Dittmann, A. , Giotopoulos, G. , Bantscheff, M. , Chan, W.‐I. , Robson, S. C. et al., Inhibition of BET recruitment to chromatin as an effective treatment for MLL‐fusion leukaemia. Nature 2011; 478: 529–533. 21964340 10.1038/nature10509 PMC3679520

[22] Bär, E. , Whitney, P. G. , Moor, K. , Reis e Sousa, C. and LeibundGut‐Landmann, S. , IL‐17 regulates systemic fungal immunity by controlling the functional competence of NK cells. Immunity 2014; 40: 117–127. 24412614 10.1016/j.immuni.2013.12.002

[23] Domínguez‐Andrés, J. , Feo‐Lucas, L. , Minguito de la Escalera, M. , González, L. , López‐Bravo, M. and Ardavín, C. , Inflammatory Ly6C high monocytes protect against candidiasis through IL‐15‐driven NK cell/neutrophil activation. Immunity 2017; 46: 1059–1072.e4. 28636955 10.1016/j.immuni.2017.05.009

[24] Domínguez‐Andrés, J. , Joosten, L. A. and Netea, M. G. , Induction of innate immune memory: the role of cellular metabolism. Curr. Opin. Immunol. 2019; 56: 10–16. 30240964 10.1016/j.coi.2018.09.001

[25] Repnik, U. , Knezevic, M. and Jeras, M. , Simple and cost‐effective isolation of monocytes from buffy coats. J. Immunol. Methods 2003; 278: 283–292. 12957415 10.1016/s0022-1759(03)00231-x

