# Supplementary material for: Bromodomain inhibitor I‐BET151 suppresses immune responses during fungal–immune interaction
Source: Eur J Immunol. 2019 Jun 21;49(11):2044–50. doi: 10.1002/eji.201848081 (PMC6899658; doi:10.1002/eji.201848081)
Supplement: Supplementary file 1 — Figure S1. Target engagement plots for GSK1210151A (I‐BET151). Figure S2. Cytotoxicity assay for I‐BET151 treatment. Figure S3. Morphological changes induced by trained immunity. Figure S4. Effects of JQ1 in the induction of trained immunity. Table 1. Primer pairs used for epigenetic study. [file EJI-49-2044-s001.pdf]

European Journal of Immunology

Supporting Information
for
DOI 10.1002/eji.201848081

Jorge Domínguez-Andrés, Anaísa V Ferreira, Trees Jansen, Nicholas Smithers, Rab K. Prinjha, Rebecca C. Furze and Mihai G. Netea

Bromodomain inhibitor I-BET151 suppresses immune responses during fungal–immune interaction

© 2019 The Authors. *European Journal of Immunology* published by WILEY-VCH Verlag GmbH & Co. KGaA, Weinheim.

Compound Number	Cytokine	Mean pIC50	SD	Range	Number of curves (n)	Mean Hill Slope	Mean asymptote max
GSK1210151A	TNFα	6,5	0,43	5.9 - 7.8	68	1,3	105
GSK1210151A	IL-6	6,1	0,36	5.5 - 7.8	116	1,1	98

% Inhibition	[GSK1210151A] M	
	TNFα	IL-6
1,25	1,10E-08	1,50E-08
2,5	1,89E-08	2,84E-08
5	3,28E-08	5,46E-08
10	5,83E-08	1,08E-07
20	1,09E-07	2,25E-07
30	1,65E-07	3,68E-07
40	2,31E-07	5,49E-07
50	3,16E-07	7,94E-07
60	4,32E-07	1,15E-06
70	6,07E-07	1,72E-06
80	9,19E-07	2,80E-06
90	1,71E-06	5,85E-06
95	3,05E-06	1,15E-05
97,5	5,30E-06	2,22E-05

Figure S1. Target engagement plots for GSK1210151A (I-BET151).

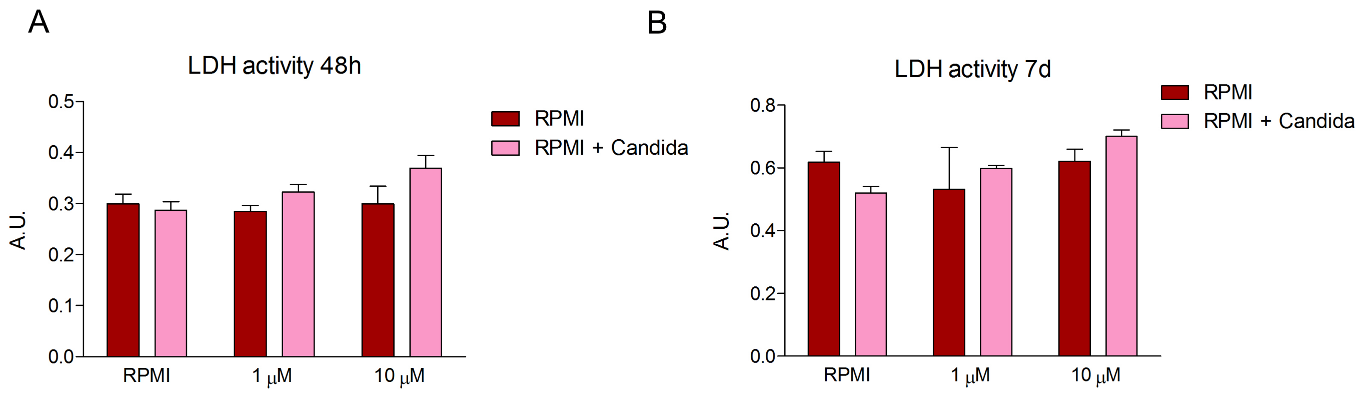

Figure S2. Cytotoxicity assay for I-BET151 treatment. Detection of cell death by lactate dehydrogenase (LDH) assay in PBMCs treated with control medium, 1 μM or 10 μM I-BET151 and stimulated with RPMI or 10^6 heat-killed *C. albicans* conidia during 48h (A) or 7 days (B) (mean ± SEM, n =3 (from 3 different individual donors)).

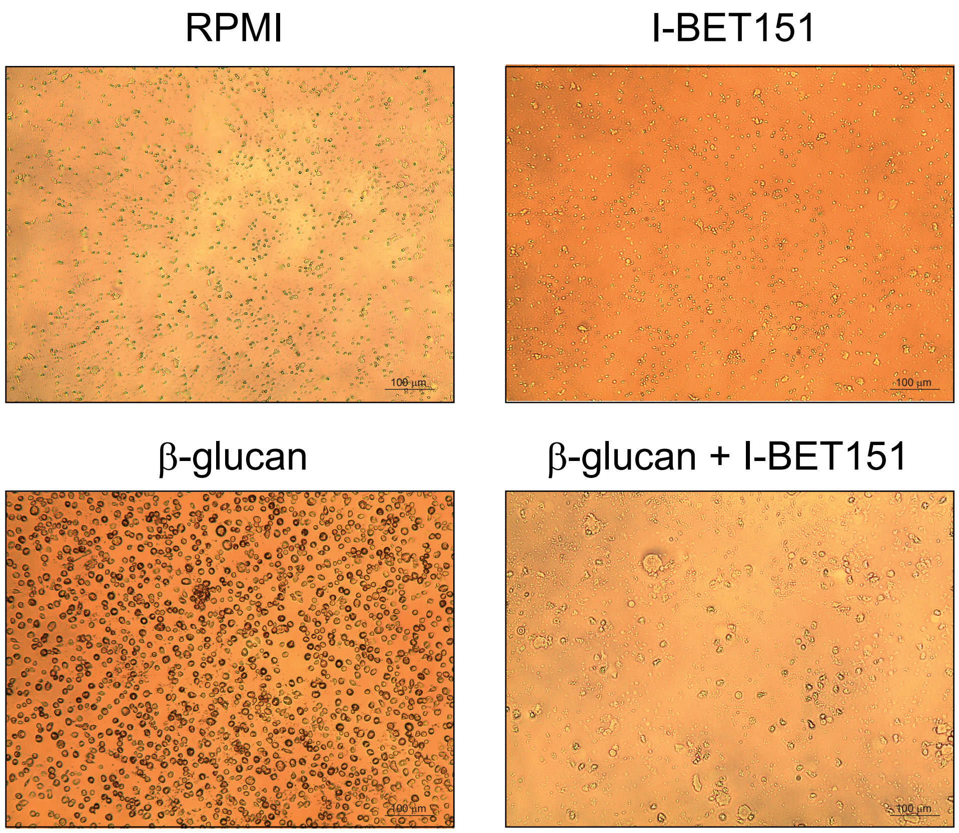

Figure S3. Morphological changes induced by trained immunity.

Morphology of cells after 24 h of training and 6 days of rest when the cells were trained with RPMI (negative control), 10 µM I-BET151, 1 mg/ml β-glucan or 10 µM I-BET151 + 1 mg/ml β-glucan. Pictures were taken at day 6, magnification x10.

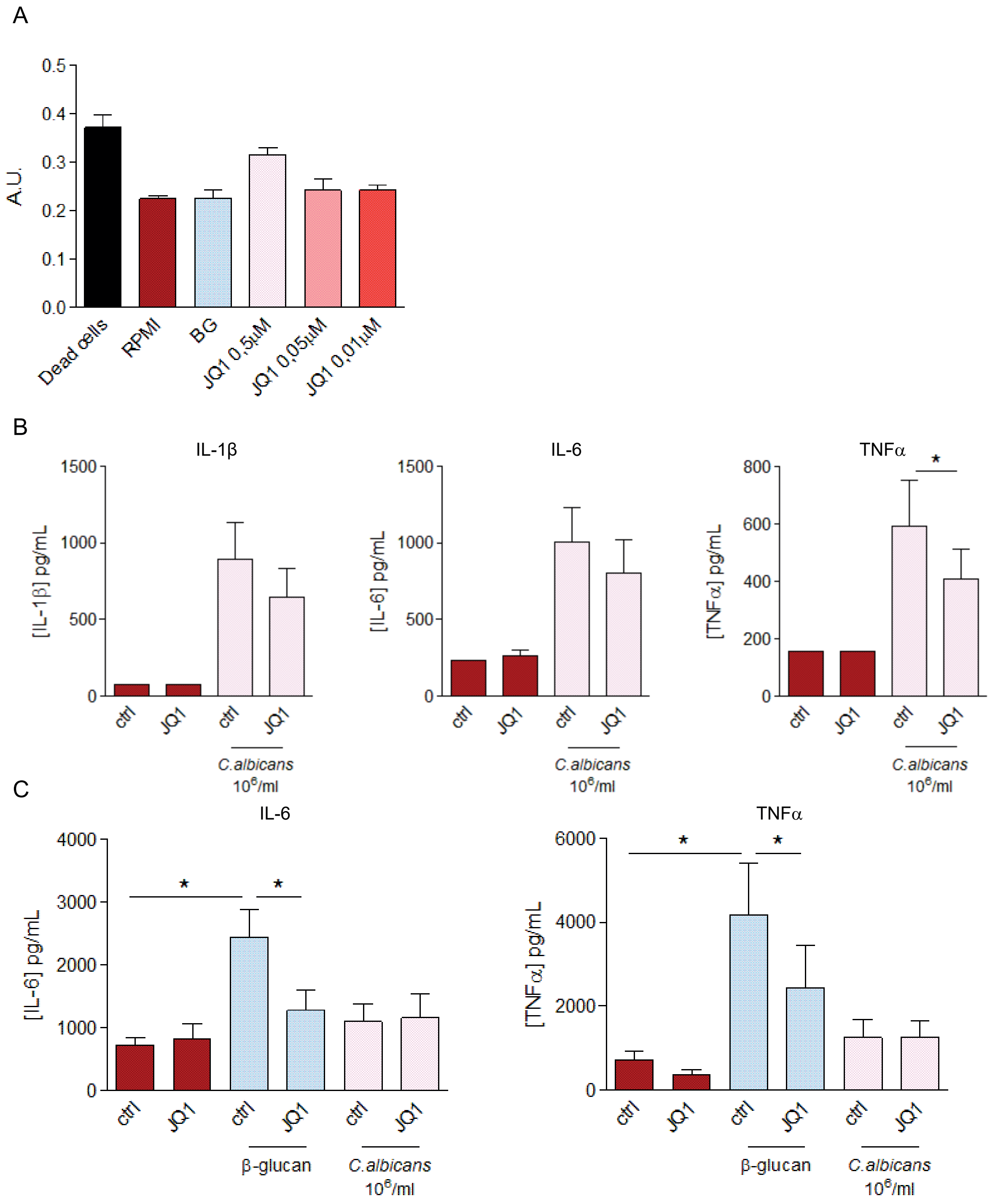

Figure S4. Effects of JQ1 in the induction of trained immunity.

(A) Cytotoxicity assay for the treatment of cells with three different concentrations of JQ1. Dead cells were used as a positive control. (B) IL-1β, IL-6 and TNFα production by human monocytes treated or not with 0.05 µM JQ1 and stimulated with 10^6/ml heat-killed *C. albicans* conidia for 24 h. (C) IL-6 and TNFα production by human monocytes treated or not with 0.05 µM JQ1 and stimulated with RPMI, 1 µg/mL β-glucan or 10^6/ml heat-killed *C. albicans* conidia, following a similar protocol displayed in Figures 3A and 3D. Cells were re-exposed to 10 ng/ml LPS after 6 days, and cytokines were measured 24 h later. Mean ± SEM, n=6 (from 6 different individual donors); pooled from 2 independent experiments with 3 individual donors each. *p<0.05, Wilcoxon signed-rank test.

Table 1. Primer pairs used for epigenetic study.

IL6.1	FW	TCGTGCATGACTTCAGCTTT
IL6.1	RV	GCGCTAAGAAGCAGAACCAC
IL6.2	FW	AGGGAGAGCCAGAACACAGA
IL6.2	RV	GAGTTTCCTCTGACTCCATCG
TNFA.1	FW	AGAGGACCAGCTAAGAGGGA
TNFA.1	RV	AGCTTGTCAGGGGATGTGG
TNFA.2	FW	GTGCTTGTTCCTCAGCCTCT
TNFA.2	RV	ATCACTCCAAAGTGCAGCAG
TNFA.3	FW	TGTCTGGCACACAGAAGACA
TNFA.3	RV	CCCTGAGGTGTCTGGTTTTC
TNFA.4	FW	AGCCAGCTGTTCCTCCTTTA
TNFA.4	RV	TTAGAGAGAGGTCCCTGGGG
TNFA.5	FW	TGATGGTAGGCAGAACTTGG
TNFA.5	RV	ACTAAGGCCTGTGCTGTTCC
TNFA.6	FW	CAGGCAGGTTCTCTTCCTCT
TNFA.6	RV	GCTTTCAGTGCTCATGGTGT

Supplementary Methods

LPS stimulated human blood assay

Blood was collected into 10 IU / mL sodium heparin and incubated for 30 min at 37ºC in a concentration response (10 µM-0.51nM) of I-BET151, prior to the addition of 200 ng/mL LPS. Supernatants were removed after 22h incubation 37ºC and analysed for IL-6 and TNFα using MesoScaleDescovery (MSD) technology. ICxx calculations were determined using the formula ICxx = ((% inhibition / (100-% inhibition))^(1/Hill Slope))*IC50).

Viability assays

Cell viability was assessed using CytoTox 96® Non-Radioactive Cytotoxicity Assay (Promega). Released LDH in culture supernatants is measured with a 30-minute coupled enzymatic assay. The amount of color formed is proportional to the number of lysed cells.
